# Tissue Transglutaminase Constitutively Activates HIF-1α Promoter and Nuclear Factor-κB via a Non-Canonical Pathway

**DOI:** 10.1371/journal.pone.0049321

**Published:** 2012-11-19

**Authors:** Santosh Kumar, Kapil Mehta

**Affiliations:** Department of Experimental Therapeutics, The University of Texas M. D. Anderson Cancer Center, Houston, Texas, United States of America; University of Nebraska Medical Center, United States of America

## Abstract

Constitutive activation of nuclear factor kappa B (NF-κB) has been linked with carcinogenesis and cancer progression, including metastasis, chemoresistance, and radiation resistance. However, the molecular mechanisms that result in constitutive activation of NF-κB are poorly understood. Here we show that chronic expression of the pro-inflammatory protein tissue transglutaminase (TG2) reprograms the transcription regulatory network in epithelial cells via constitutive activation of NF-κB. TG2-induced NF-κB binds the functional NF-κB binding site in hypoxia-inducible factor-1 (*HIF-1α*) promoter and results in its increased expression at transcription and protein levels even under normoxic conditions. TG2/NF-κB-induced HIF-1 was deemed essential for increased expression of some transcription repressors, like *Zeb1*, Zeb2, *Snail*, and *Twist*. Unlike tumor necrosis factor-alpha (TNFα), TG2 did not require IκB kinase (IKK) for NF-κB activation. Our data suggest that TG2 binds with IκBα and results in its rapid degradation via a non-proteasomal pathway. Importantly, the catalytically inactive (C277S) mutant form of TG2 was as effective as was wild-type TG2 in activating NF-κB and inducing HIF-1 expression. We also found that TG2 interacted with p65/RelA protein, both in the cytosolic and the nuclear compartment. The TG2/p65(NF-κB) complex binds to the *HIF-1* promoter and induced its transcriptional regulation. Inhibition of TG2 or p65/RelA also inhibited the HIF-1α expression and attenuated *Zeb1, Zeb2*, and *Twist* expression. To our knowledge, these findings show for the first time a direct link between TG2, NF-κB, and HIF-1α, demonstrating TG2's important role in cancer progression.

## Introduction

Tumor progression and metastasis require cancer cells to circumvent stressful environments, such as those lacking adequate oxygen or nutrients. Such environments promote the metastatic potential of cancer cells by activating cell survival mechanisms against proapoptotic signals induced by physiological and pharmacological agents (including chemotherapeutic drugs). Over the past two decades, many genes and pathways have been identified that may be involved in drug resistance [1]. Among these, nuclear factor kappa B (NF-κB) activation has been strongly implicated in chemoresistance and metastasis [2], [3]. Thus, agents that downregulate NF-κB should sensitize tumors to chemotherapy and prevent metastasis [4].

Although NF-κB activity is considered essential for mediating innate and humoral immune responses, its activation in non-immune cells can be deleterious to the host. In normal cells NF-κB exists in inactive form but almost all cancer cell types exhibit constitutive activation of NF-κB [5]. Inhibition of NF-κB in a tumor-specific manner could have enormous therapeutic potential, but if NF-κB is inhibited non-specifically, the effects could be devastating for normal immune response [6]. The mechanisms that underlie constitutive activation of NF-κB in cancer cells are not fully understood, but they are thought to be different from inducible activation [5]. Therefore, information on pathways that result in constitutive activation of NF-κB may offer promising therapeutic targets for selectively inhibiting this transcription factor. Here, we provide evidence that aberrant expression of the pro-inflammatory protein tissue transglutaminase (TG2), induces constitutive activation of NF-κB owing to TG2's interaction with and rapid degradation of the inhibitory protein IκBα.

TG2 is structurally and functionally a complex protein encoded by the gene *TGM2,* located on chromosome 20 (20q11.2–q12) [7], [8]. *TGM2* is considered to be a stress-responsive gene, and its expression is upregulated in response to stressors such as tissue injury, inflammatory cytokines, UV radiations, and reactive oxygen species [9]. TG2 expression is involved in restoring normal homeostasis at the site of injury by stabilizing the extracellular matrix (ECM) [8]. Several reports have documented increased expression of TG2 in multiple cancer cell types and TG2 expression is associated with poor disease outcome [10]–[14]. Increase in TG2 expression is frequently observed during advanced stages of disease, metastatic spread, and drug resistance [10]–[19]. Recently, we found that aberrant expression of TG2 is sufficient to induce transdifferentiation of mammary epithelial cells into mesenchymal cells, a process known as epithelial-to-mesenchymal transition (EMT) [20]. EMT, an embryonic development process, is frequently reactivated in cancer cells and is thought to have a role in tumor aggressiveness and metastasis [21], [22]. TG2 expression in ovarian cancer cells also induced EMT, as characterized by the loss of epithelial markers and by the increased ability of cells to form tumors, peritoneal metastasis, and malignant ascites in an orthotopic model [23]. Importantly, the TG2-induced EMT was associated with NF-κB activation in both mammary and ovarian cancer cells [23], [24].

Both TG2 and NF-κB have been implicated in inflammation-induced signaling and in progression of cancer [9], [25]–[27], and both can induce EMT [23]–[25], [28], [29]. On the basis of these observations, we reasoned that TG2 could induce EMT and promote an aggressive phenotype by activating NF-κB. Here, we report a novel TG2-regulated pathway that constitutively activates NF-κB and increases the transcriptional regulation of the *HIF-1α* gene, thus linking these important pathways in a common mechanism induced by aberrant expression of TG2 in cancer cells.

## Results

### Crosslinking Activity of TG2 is not Essential for NF-κB Activation

**Figure 1:**
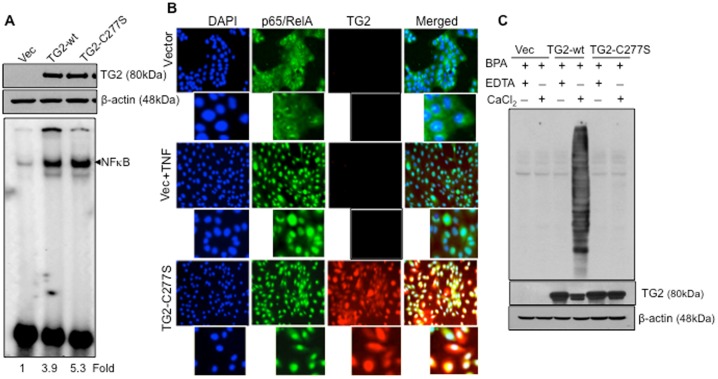
Catalytic function of TG2 is not essential for activation of NF-κB. A- Western blot analysis for TG2 expression in MCF10A cells stably transfected with either a lentiviral construct alone (Vec), or a lentiviral containing a TG2-wt or a TG2-C277S construct (upper frame). The membrane was stripped and reprobed with anti-β actin antibody to ensure even protein loading in each lane (middle frame). Electrophoretic mobility shift assay (EMSA) was performed using ^32^P end-labeled 45-mer double-stranded NF-κB oligonucleotide probe (5′-TTG TTA CAA GGG ACT TTC CGC TGG GGA CTT TCC AGG GAG GCG TGG-3′) using nuclear extracts prepared from vector, TG2-wt or TG-C227S-transfected MCF10A cells (lower panel). B- Localization of p65/RelA (green) protein in nuclei (blue) of vector alone or after their stimulation with TNFα (10 ng/ml for 30 minutes) and TG2-C2277S-transfected MCF10A cells, as determined by an immunofluorescence assay. TG2 was stained with PE-conjugated secondary antibody (red). Magnification- ×100 original, ×500 magnified view. C- Cross-linking activity of endogenous TG2 was determined by BPA conjugation using total cell extracts prepared from MCF10A cells transfected with vector alone or with TG2-WT or TG2-C277S. The BPA conjugation was determined in the presence (+CaCl_2_) or absence (+EDTA) of 5 mM Ca^2+^, as described in the Materials and Methods section. Fifteen micrograms of protein from each reaction mixture was subjected to Western blot analysis to determine TG2 and β actin levels (lower panel). Results shown are from a representative experiment, repeated at least twice with similar results.

Overexpression of TG2 in various cell types is associated with the constitutive activation NF-κB [30]–[34]. The TG2-catalyzed polymerization of IκBα, which renders IκBα unable to bind and sequester NF-κB in the cytosol, was proposed to be the mechanism responsible for this effect [31]. However, the evidence that low Ca^2+^ and high GTP levels are unlikely to allow intracellular TG2 to acquire extended or catalytically active conformation [35] strongly argues against such a hypothesis. To address this question, we first determined the ability of the catalytically inactive mutant form of TG2 (TG2-C277S) to induce NF-κB activation. MCF10A cells were stably transfected with wild-type (TG2-wt) or catalytically inactive TG2 (TG2-C277S) (Figure 1A). Results shown in Figure 1 demonstrate that, irrespective of the catalytic activity, TG2 expression resulted in activation of NF-κB. Thus, MCF10A cells expressing either catalytically active (TG2-wt) or inactive (TG2-C277S) TG2 showed a significant increase in NF-κB activation as revealed by EMSA. Immunofluorescence staining confirmed the EMSA results and supported constitutive activation of NF-κB in TG2-C277S-expressing cells (Figure 1B). As expected, the cytospin preparations of control vector-transfected cells showed no nuclear staining for p65/RelA. However, following their treatment with TNFα (10 ng/mL for 30 minutes), more than 70% of cells became positive for p65/RelA nuclear staining. In contrast, the majority of TG2-C277S-expressing cells (more than 90%) showed strong p65/RelA nuclear staining without any TNFα treatment. The p65/RelA protein appeared to co-localize with TG2 in the nuclei of these cells as revealed by strong yellow fluorescence in merged images (Figure 1B). To further establish that the retarded band visualized by EMSA in the TG2-C277S-expressing cells was indeed NF-κB, we incubated nuclear extracts from these cells with antibodies to the p65/RelA subunit and then conducted EMSA. Antibodies to p65/RelA decreased the DNA binding (Figure S1), suggesting that the TG2-activated complex includes p65/RelA. Pre-immune serum had no affect on the mobility of NF-κB. Excess unlabeled NF-κB (100-fold) caused the complete disappearance of the band, but mutant oligo did not, indicating the specificity of NF-κB. Anti-TG2 antibody failed to cause a shift in the NF-κB band, probably owing to the high salt conditions employed for preparing the nuclear extract that might cause dissociation of TG2 from the p65/RelA complex.

Next, we established that the TG2-C277S protein in MCF10A cells was indeed catalytically inactive (Figure 1C). For this purpose, transamidation activity in cell lysates was determined by studying the conjugation of BPA to cellular proteins in the presence or absence of Ca^2+^. As expected, no BPA conjugation, regardless of the presence or absence of Ca^2+^ was evident in cell extracts lacking TG2 expression (Vector-transfected) or harboring the catalytically inactive mutant form of the TG2 protein (TG2-C277S). TG2-WT-transfected cells, in contrast, showed significant conjugation of BPA to multiple cellular proteins only in the presence of Ca^2+^ (Figure 1C). These results affirmed that the TG2-WT protein requires high Ca^2+^ to become catalytically active, whereas the TG2-C277S protein is deficient in transamidation activity even in the presence of high Ca^2+.^

### TG2-Induced NF-κB is not Cell-Type Specific

To determine whether TG2 can activate NF-κB in other cell types, the nuclear extracts from the MCF-7 breast cancer cell line and its drug-resistant sublines thereof (MCF7/RT and MCF-7/DOX) were analyzed for NF-κB activity. The EMSA results shown in Figure S2A demonstrate constitutive activation of NF-κB only in cells with constitutive (RT, DOX) or induced TG2 (TG2-WT, TG2-C277S) expression. Another mutant form of TG2 (TG2-R580A), which retains the transamidation activity but lacks a guanine nucleotide (GTP)-binding function [24], was relatively week in inducing NF-κB activation in MCF10A cells (Figure S2A). TG2-induced activation of NF-κB was associated with an increase in p65 phosphorylation (Figure S3). These results support the conclusion that the aberrant expression of TG2 in transformed or non-transformed mammary epithelial cells is associated with the constitutive activation of NF-κB.

**Figure 2:**
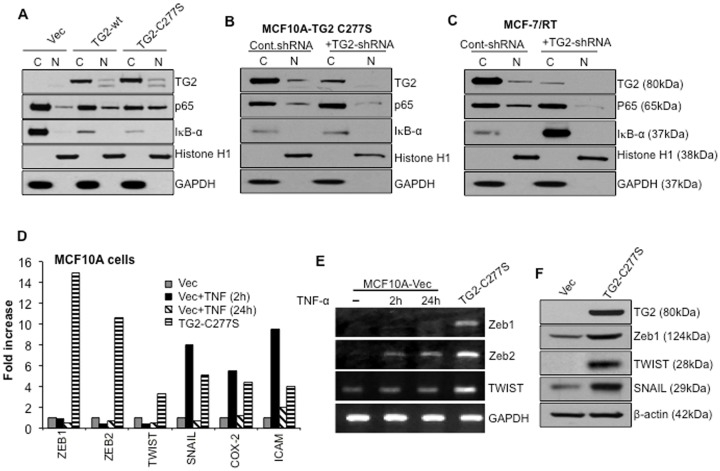
TG2 expression correlates with constitutive NF-κB activation. A- Western blot analyses of nuclear and cytosolic fractions prepared from vector, TG2-WT, and TG2-C277S-expressing MCF10A cells. Membranes were probed with either anti-TG2 or anti-p65/RelA antibodies, stripped, and reprobed with anti-IκBα, GAPDH or anti-histone antibodies to determine even protein loading and purity of cytosolic and nuclear fractions, respectively. The low IκBα protein levels in cytosolic fractions were related to TG2 expression, as suggested by the even β actin band in all the three cytosolic fractions (data not shown). B- Detection of p65/RelA in the nuclear fraction of TG2-C277S transfected MCF10A cells and C- in MCF-7/RT cells after transfection with either the control or TG2-specific shRNA. Membranes were reprobed with anti-IκBα, GAPDH or anti-histone antibodies to ensure the even loading and purity of cytosolic and nuclear fractions. D- Quantitative RT-PCR array showing relative changes in the expression of NF-κB target genes in TG2-C277S-transfected MCF10A cells and MCF10A-vector cells after stimulation with TNFα (10 ng/mL) for the indicated times. The Y-axis denotes the fold-expression, and the x-axis denotes the genes. Expression of GAPDH, β actin, and 18S ribosomal RNA was used to normalize the variable template loading. E- RT-PCR analysis for basal and TNFα-induced expression of selected NF-κB target genes in TG2-C277S and control vector-transfected MCF10A cells. F- Immunoblot analysis for basal expression of indicated NF-κB target genes in TG2-C277S and vector-transfected MCF10A cells. Results shown are from a representative experiment repeated at least 2 or 3 times with similar results.

We further validated TG2-C277S-induced NF-κB activation by studying the nuclear localization of the p65/RelA subunit of NF-κB in different cell types. Results shown in Figure 2A reveal the presence of the p65/RelA band in nuclear extracts isolated from cells expressing TG2-C277S and TG2-wt; vector-infected cells showed relatively weak bands. Interestingly, we also observed that the basal level of the IκBα protein in TG2-expressing cells was significantly lower than in vector infected control cells (Figure 2A). To further validate these results, we downregulated TG2 expression in MCF10A/TG2-C277S (Figure 2B) and MCF-7/RT cells (Figure 2C) using TG2-specific shRNA. The nuclear and cytosolic extracts from TG2-inhibited and control cells were studied for p65/RelA and IκBα expression. Results shown in Figure 2B and 2C demonstrate - attenuation of the p65/RelA localization in the nucleus. Notably, TG2 downregulation caused a slight but reproducible increase in IκBα in TG2-C277S-expressing cells but caused a significant increase in MCF-7/RT cells. These results suggest that, irrespective of its catalytic function, TG2 expression results in the constitutive activation of NF-κB and that this effect might be related to TG2's ability to modulate IκBα level.

### TG2-Induced NF-κB Target Genes

Next, we compared the NF-κB target gene profiles induced in response to TG2 expression (constitutive) or TNFα treatment (inducible). A real-time PCR-based array of 13 known NF-κB target genes was designed (SABioscience), and the basal and TNFα-induced transcript levels in the vector- and TG2-C277S-transfected MCF10A cells were determined (Table S1). Results shown in Figure 2D reveal a strong and selective increase in the Zeb1 and Zeb2 transcript in TG2-C277S-expressing cells. Expression of other NF-κB target genes (*Twist, Snail, Cox 2,* and *ICAM*) in these cells was moderate. On the other hand, TNFα-induced activation of NF-κB (after 2 hours of treatment) caused a noticeable increase in *Snail, Cox-2* and *ICAM* transcripts, whereas *Zeb1, Zeb2* and *Twist* levels remained the same. The TNFα-induced alterations in gene expression were transient, as the transcript levels returned to a basal level after 24 hours. The TG2-C277S and TNFα-induced changes in some selected target gene expressions were further validated by RT-PCR (Figure 2E) and Western blotting (Figure 2F), and these results further supported the hypothesis that TG2-C277S-expressing cells contain higher basal levels of *Zeb1, Zeb2, Snail* and *Twist.*

**Figure 3:**
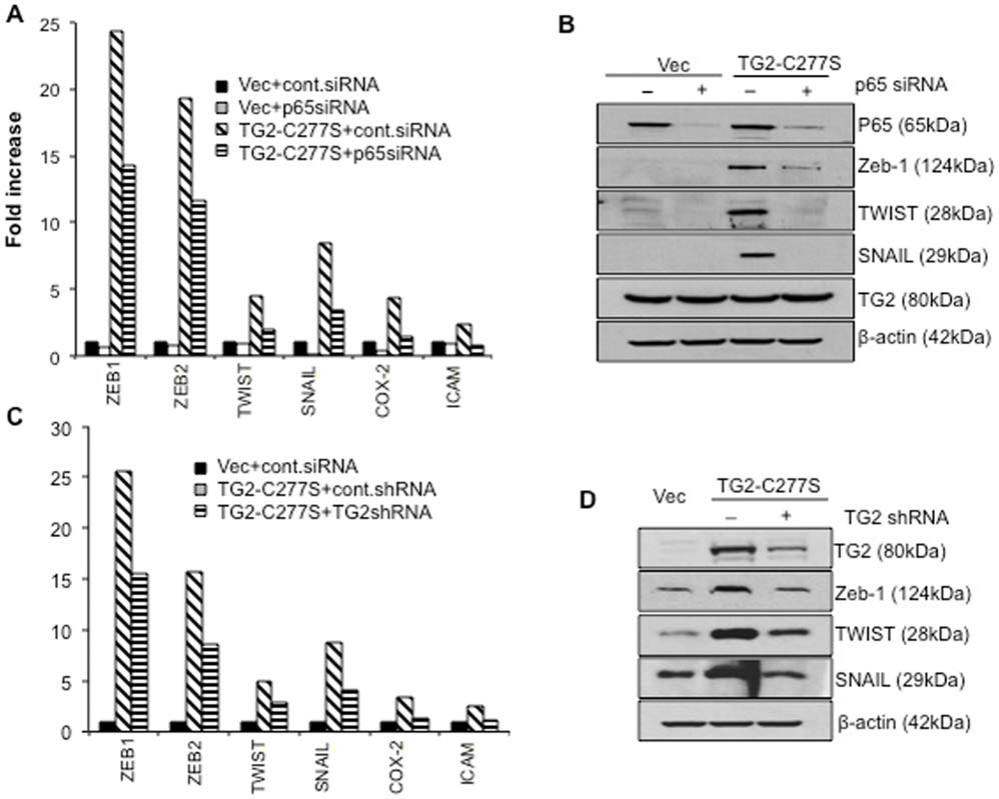
TG2-induced expression of transcription repressor genes is mediated though NF-κB. A- Quantitative RT-PCR array showing relative changes in the expression of NF-κB related genes in TG2-C277S and vector-transfected MCF10A cells before and after transfection with control or p65/RelA - siRNA. B- Immunoblot analysis of NF-κB target genes in cells transfected with TG2-C277S or the vector alone before and after transfection with control or p65/RelA- siRNA. Membranes were reprobed with anti-TG2 and β-actin antibodies to ensure, if any, the effect of p65/RelA downregulation of TG2 expression and even protein loading in each lane. C- Quantitative RT-PCR array showing relative changes in the expression of NF-κB responsive genes- in vector-control andTG2-C277S MCF10A cells in response to TG2 downregulation. The y-axis denotes the fold- expression and the x-axis denotes the genes. The expression of GAPDH, β-actin, and 18S ribosomal RNA was used to normalize variable template loadings. D- Immunoblot analysis was performed to validate the effect of TG2 downregulation on NF-κB target genes in TG2-C277S MCF10A cells. The membrane was reprobed with anti-β-actin to ensure equal protein loading. Results shown are from representative experiments repeated at least 3 times with similar results.

To determine whether high basal levels of *Zeb1, Zeb2, Snail* and *Twist* in TG2-C277S- expressing cells is due to the constitutive activation of NF-κB, we next tested the effect of NF-κB inhibition on these transcripts. The vector- and TG2-C277S-expressing cells were transfected with control- or p65/RelA-specific siRNA, and the expression of NF-κB target genes was determined. We were able to downregulate p65/RelA expression by 60–70% after 72 hours of transfection with siRNA. No noticeable change in TG2-C277S expression was observed under these conditions. The real-time PCR and immunobltting data shown in Figure 3A and 3B, respectively, suggest that downregulation of p65/RelA in TG2-C277S-expressing cells is associated with attenuation in target gene expression. Data shown in Figure 3C and 3D reveal that down regulation of TG2 reduces the levels of NF-κB target gene expression. This effect was confirmed in the MFC-7/RT cells, which express high basal levels of catalytically active TG2. Results shown in Figure S4 further established that the inhibition of TG2 is associated with the downrgulation of target gene expression. Taken together, these results suggest that the TG2-regulated activation of NF-κB is essential for the sustained expression of target genes, including *Zeb1, Zeb2, Twist* and *Snail*, the transcription repressors that promotes an EMT phenotype.

### TG2-Induces NF-κB Activation via Non-Canonical Pathway

**Figure 4:**
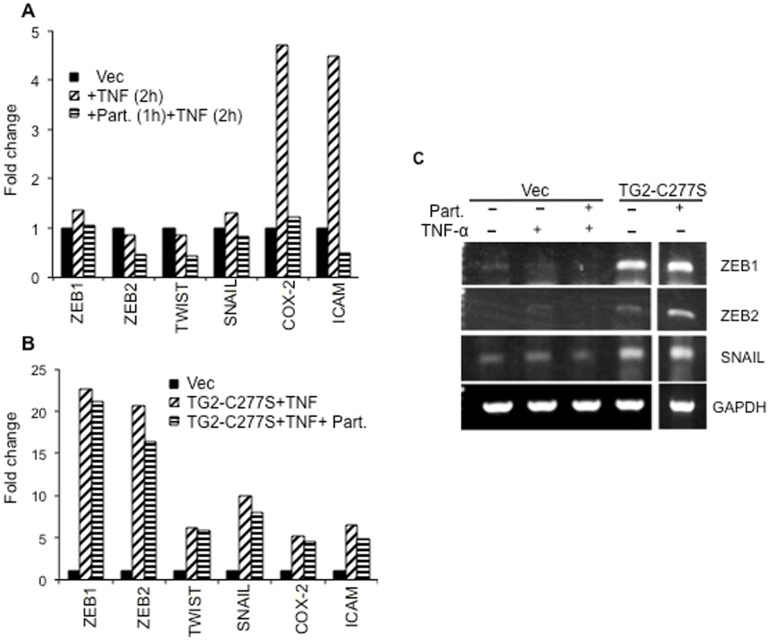
TG2-induced NF-κB activation is independent of IKK activity. A- Quantitative RT-PCR array showing relative changes in the expression of NF-κB target genes in response to TNFα (10 ng/mL, 2 hours) treatment and the effect of IKK inhibition (parthenolide, 5 µM) on TNFα induced gene expression in MCF10Avec cells. B- Quantitative RT-PCR analysis of NF-κB responsive genes in TG2-C277S MCF10A cells incubated with TNFα (10 ng/mL, 2 hours) in the presence or absence of parthenolide. C- RT-PCR analysis was performed to validate the expression of Zeb1, Zeb2 and Snail1 in MCF10A-Vec cells incubated with TNFα (10 ng/ml, 2 hours) and TG2-C277S MCF10A cells in the presence or absence of parthenolide (5 µM). Results shown are from a representative experiments repeated at least twice with similar results.

Next, we determined whether TG2 regulates NF-κB activation via a canonical pathway. MCF10A cells transfected with a control (vector) or TG2-C277S were preincubated with the IκB kinase inhibitor parthenolide prior to their treatment with TNFα. Analysis of NF-κB responsive genes revealed that preincubation of vector-transfected cells with parthenolide could completely inhibit TNFα-induced gene expression (Figure 4A). TG2-C277S MCF10A cells, in contrast, showed no or moderate change in target gene expression in response to parthenolide treatment (Figure 4B). The effect of parthenolide treatment on *Zeb1, Zeb2*, and *Snail* transcript expression was further validated by RT-PCR (Figure 4C). These results suggest that the TG2-induced activation of NF-κB is not dependent on the IKK pathway.

### TG2-Induced NF-κB Activation due to Downregulation of IκBα

**Figure 5:**
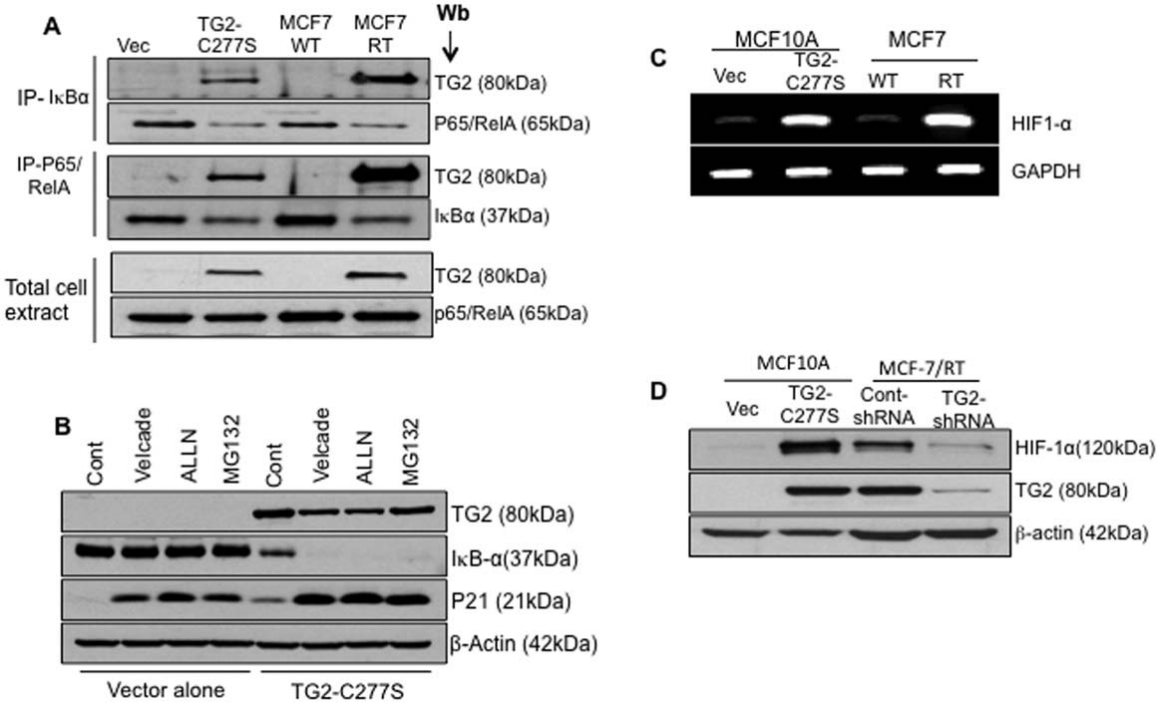
TG2 mediate IκBα degradation via non-proteasomal pathway and results in HIF-1α expression. A- Total cell extracts prepared from MCF10A-Vec, MCF10A TG2-C277S, drug-sensitive (MCF-7/WT) and drug resistant (MCF-7/RT) MCF-7 cell were immunoprecipitated with anti-p65/RelA or anti-IκBα antibody and immunoblotted with anti-TG2, anti- p65/RelA, or anti- IκBα antibody. Total cell extracts (15 µg protein) were also directly subjected to immunoblotting to determine TG2 and p65/RelA levels. B- MCF10A-Vec and MCF10A TG2-C277S cells were incubated with medium alone (Cont.) or medium containing the indicated proteasomal inhibitor [Bortezomib (20 nM), N-acetyl-leucyl-leucyl-norleucinal (ALLN) (50 µg/mL), and MG-132 (1 µM)] for 2 hours. At the end of incubation, cells extracts were prepared and subjected to immunoblot analysis, using anti-TG2, anti-IκBα and anti-p21 antibody (p21 served as a positive control) and β-actin to ensure equal protein loading. C- RT-PCR analysis showing the basal transcript level of HIF-1α in MCF10A-Vec, MCF10A TG2-C277S, MCF7-WT and MCF7/RT cells. Results shown are from a representative experiment repeated at least 2 times with similar results. D- Immunoblot analysis showing the basal HIF-1α and TG2 protein expression in MCF10A-Vec, MCF10A TG2-C277S, MCF7-WT and MCF7/RT cells under normoxic conditions.

On the basis of the observation that TG2-C277S expression is associated with decreased levels of IκBα in mammary epithelial cells (Figure 2), we reasoned that TG2 may affect IκBα stability. To test this theory, we first determined whether TG2 interacts with IκBα. Cytosolic extracts from TG2- expressing (TG2-C277S MCF10A and MCF-7/RT) and TG2-deficient (MCF10A-Vec and MCF-7/WT) cells were immunoprecipitated with anti- IκBα or p65/RelA antibodies and the immunoprecipitates were subjected to immunoblotting with anti-TG2 antibodies. Results shown in Figure 5A clearly demonstrated that TG2 is associated in complex with IκBα and p65/RelA, as revealed by the distinct TG2 pulled-down band in the IκBα and p65/RelA immunoprecipitates. To determine the fate of the TG2/IκBα complex in MCF10A cells, we next studied the effect of 3 different proteasomal inhibitors, with surprising results. Inhibition of the proteasomal pathway did not cause any noticeable change in IκBα levels in the control vector-infected cells, whereas TG2-C277S-expressing cells showed almost a complete loss of the IκBα protein under identical conditions (Figure 5B). The TG2 level remained unchanged in the presence or absence of proteasomal inhibitors. As a control, we also determined the cyclin-dependent kinase inhibitor 1 (p21) levels in the same samples and observed a remarkable accumulation in p21 levels in inhibitor-treated cells both in TG2-deficient (vector alone) and in TG2-expressing (TG2-C277S) MCF10A cells (Figure 5B). These results suggest that TG2 promotes destabilization/degradation of the IκBα protein in the proteasomal-independent pathway and that inhibition of the proteasomal pathway further accelerates TG2-mediated degradation of IκBα.

### TG2-NF-κB Binds and Activates an *HIF-1α* Promoter

Because the crosstalk between the NF-κB pathway and the HIF pathway has been well- documented [36] we next explored whether TG2-induced NF-κB activation could modulate HIF-1 expression. Results shown in Figure 5C reveal a considerable increase in the *HIF1α* transcript levels in TG2-overexpressing cells, both in induced (TG2-C277S in MCF10A cells) and with constitutive (in MCF-7/RT cells) TG2 expression. This increase in transcript level paralleled an increased accumulation of HIF-1α protein in TG2-expressing cells even under normoxic conditions (Figure 5D). Furthermore, downregulation of TG2 by shRNA in MCF-7/RT cells reduced HIF-1α protein expression by 70–80% (Figure 5D). These results suggested that TG2-induced activation of NF-κB may play a role in the transcriptional regulation of *HIF-1*.

**Figure 6:**
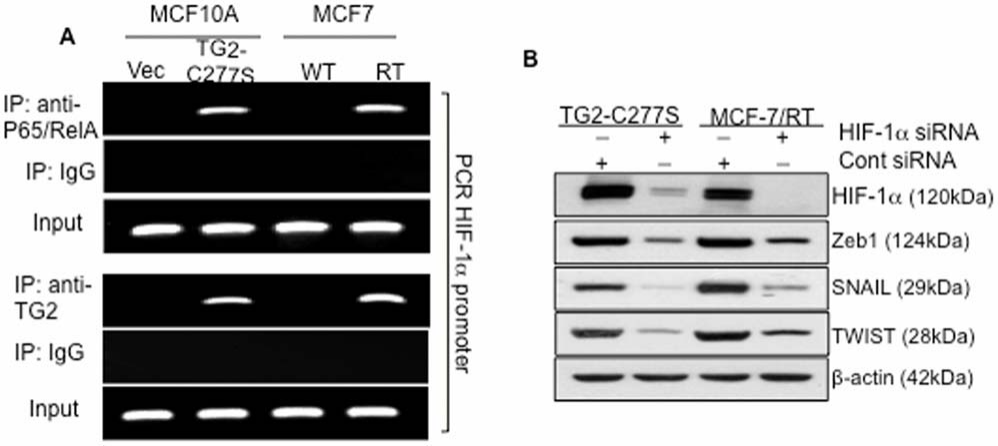
Chromatin immunoprecipitation (ChIP) showing direct binding of p65/RelA in complex with TG2-C277S protein to the HIF-1α promoter. A- ChIP assay was performed on formaldehyde fixed MCF10A-Vec, MCF10A TG2-C277S, MCF7-WT, and MCF7/RT cell extracts using p65/RelA (upper panel), TG2 (lower panel), or the control anti-mouse IgG antibodies. PCR reaction was performed on immunoprecipitates, using a specific set of primers (shown in the Materials and Methods section) to amplify HIF-1α promoter sequence containing the NF-κB binding site (−197/−188 bp). Prior to immunoprecipitation, an aliquot from each sample was saved to determine the ‘input’ representing the PCR amplification of 1% of the genomic DNA without immunoprecipitation (PCR control). The sonicated DNA fraction from each sample was also subjected to PCR reaction (data not shown. B- Immunoblot analysis of Zeb1, Snail, and Twist protein expression in HIF-1α downregulated MCF10A TG2-C277S and MCF-7/RT cells. Membranes were stripped and reprobed with anti- HIF-1α antibody to determine the extent of HIF-1α inhibition by gene-specific or control siRNA and with anti β-actin antibodies to ensure even protein loading in each lane.

To test this possibility, we used a ChIP assay to study the binding of p65/RelA to the functional NF-κB binding site located at −197/−188 bp of the *HIF-1α* promoter [37] and detected the binding of p65/RelA to *HIF-1α* promoter (Figure 6A, upper panel). Cells lacking TG2 expression (MCF10A vector and MCF-7/WT) failed to show this binding activity. On the basis of the observation that TG2 co-localized with p65/RelA in the nucleus (Figure 2) and also formed a complex in the cytosolic compartment (Figure 5A), we tested whether p65/RelA binds to the *HIF-1α* promoter with TG2. The ChIP assay was performed with antibodies to TG2, and binding to *HIF-1α* promoter was detected by PCR using primers flanking the NF-κB binding site (−197/−188 bp). Results shown in Figure 6A (lower panel) clearly revealed that TG2 could pull-down the same *HIF-1α*promoter sequence as did the p65/RelA, suggesting that p65/RelA binds to the *HIF-1α* promoter in complex with TG2. No such binding activity of p65/RelA or TG2 was observed in TG2-deficient cells or in control IgG chromatin immunoprecipitates. The involvement of TG2-induced NF-κB in regulating *HIF-1α* expression was further validated by inhibiting p65/ReA expression. Downregulation of p65/RelA, using the siRNA approach, caused a significant decrease in HIF-1α protein levels without altering TG2 expression (data not shown).

**Figure 7:**
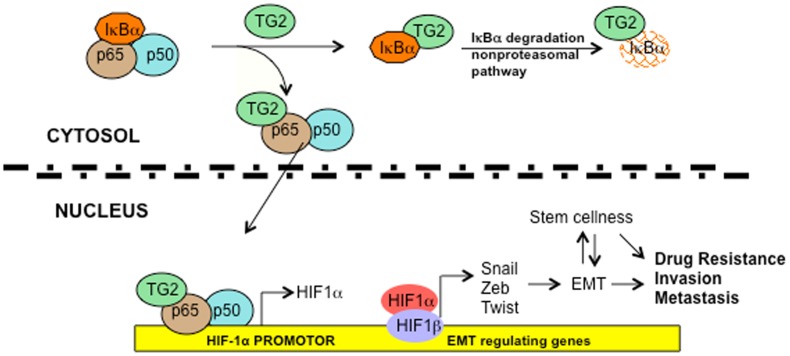
Schematic representation of TG2-regulated signaling. Association of TG2 with IκBα results in its rapid degradation via non-proteasomal pathway and liberates the p65/RelA:p50 NF-κB complex. TG2 also associates in complex with the p65/RealA subunit of NF-κB and translocates to the nucleus where it binds to the cognate NF-κB binding site on the HIF-1α promoter and results in its transcription regulation. Increased expression of HIF-1α under normoxic conditions results in increased expression of various downstream target genes such as transcription repressors *Zeb1, Zeb2, Snail1* and *Twist*, which induce EMT and promote cell survival, invasiveness, and cellular plasticity. Overall, these TG2-regulated changes contribute to increased chemoresistance and metastatic potential.

We previously reported that TG2 expression in mammary epithelial cells is associated with EMT and stem cellness [20], [24]. To determine whether TG2 mediates these events via NF-κB/HIF-1α activation, we next tested the effect of HIF-1α downregulation on the expression of EMT mediators Snail, Twist, and Zeb1 in TG2-expressing cells. Results shown in Figure 6B demonstrated that inhibition of HIF-1α in TG2-expressing cells caused marked diminution in the expression of these transcription repressors. On the basis of our earlier results, which suggested that downregulation of TG2 [20] or NF-κB [24] results in a similar inhibition of transcription repressors and reversal of EMT we propose that aberrant expression of TG2 in cancer cells promotes chemoresistance and an invasive phenotype owing to the constitutive activation of NF-κB and HIF-1α (Figure 7).

## Discussion

In this study we demonstrated that aberrant expression of the proinflammatory protein TG2 plays a central role in promoting an aggressive phenotype in cancer cells by orchestrating transcriptional regulation. Thus, ectopic or constitutive expression of TG2 resulted in the sustained activation of NF-κB via a non-canonical pathway owing to its binding to and rapid degrading of the IκBα protein. TG2-induced NF-κB upregulates the expression of another important transcription factor, HIF-1α by binding to a functional NF-κB binding site in the *HIF-1α* promoter. TG2/NF-κB-induced HIF1 expression, in turn, was deemed necessary for high basal expression of the transcription repressors such as Twist, Snail, and Zeb1, the known inducers of EMT and stem cellness in cancer cells [38]–[40]. Indeed, previous studies have suggested that TG2 expression in multiple cancer cell types is associated with both EMT and the stem cell phenotype [20], [23], [24], [41]. Another important finding of this work is our observation that the catalytic function of TG2 (transamidation activity) is not essential for NF-κB activation, a finding supported by recent observations suggesting that catalytically inactive mutant forms of TG2 (C277S and W241A) are as effective as the wild-type TG2 in inducing EMT, stem cellness, drug resistance, and an invasive phenotype in cancer cells [24].

It is now well-accepted that transformed cells can reactivate the embryonic program of epithelial plasticity to undergo EMT, enabling them to switch from a sessile, epithelial phenotype to a motile, mesenchymal phenotype [21], [22]. Although, we know EMT plays an indispensible role in tumor invasion and metastasis, we still do not understand the signaling pathways that initiate EMT in cancer cells. The major difference between normal and cancer epithelial cells is that cancer cells often become autonomous and are less affected by paracrine or negative feedback mechanisms that normally prevent excessive migration, dissemination, and organ colonization. Therefore, tumors have been often referred to as the corrupt system of normal developmental processes, and EMT is considered to be the most common fatal consequence in tumorigenesis [21], [22]. Several studies have also indicated a link between chronic inflammation and cancer progression [43], especially the constitutive activation of the inflammatory transcription factor NF-κB has been implicated to play a central role in inflammation-induced progression of cancer [28], [29]. For example, Huber et al. [28] reported that NF-κB is the key mediator of EMT in an animal model of breast cancer progression. Thus, inhibition of NF-κB reduced metastases to the lungs of mice by a factor of 10 and decreased tumor weight in the mammary fat pad model by a factor of 3.

Two independent pathways regulate NF-κB activation. The canonical pathway, which involves NF-κB activation in response to various inflammatory stimuli (such as tumor necrosis factor-α [TNFα], interleukin-1 [IL-1], and bacterial products), is activated through the IKKα/IKKβ/IKKγ complex [6]. Upon stimulation, the activated IKKβ phosphorylates IκBα at Ser32 and Ser36 resulting in its rapid degradation via 26S proteasome proteolysis and the consequent release of NF-κB. NF-κB heterodimer then translocates to the nucleus, binds to its cognate DNA motifs in the promoter, and induces the expression of target genes. Interestingly, one of the early response genes for activated NF-κB is IκBα, which then binds back to NF-κB and silences its activation. This feedback loop is probably essential for preventing the long-term activation of NF-κB in normal cells. In contrast, most cancer cells exhibit constitutive activation of NF-κB via a noncanonical pathway [26], which in turn activates multiple downstream target genes implicated in cell survival, cell proliferation, angiogenesis, and metastasis [2]–[4]. Despite frequent observation of constitutive activation of NF-κB in advanced stage tumors, exact mechanism of NF-κB activation in cancer cells remains largely unknown.

Many recent studies support that the aberrant expression of TG2 can produce oncogenic signaling that promotes an aggressive phenotype in cancer cells [15]–[20], [44] and thus may represent the missing link between inflammation and cancer progression. Indeed, increased expression of TG2 is associated with NF-κB activation in breast cancer cells selected for resistance to doxorubicin [31]. TG2 catalyzes crosslinking of IκBα, resulting in a polymer formation of these proteins that is unable to bind and sequester NF-κB in the cytosol [34]. Similarly, TG2-induced NF-κB activation was implicated in resistance to cisplatin in ovarian cancer cells, and the synergistic interaction between TG2 inhibitor KCC009 and cisplatin suggests that the transamidation activity of TG2 is essential for NF-κB activation [41], [42]. More recently, Jung et al. [45] reached a similar conclusion and suggested that TG2-induced resistance to bortezomib in mantle cell lymphoma is mediated via NF-κB activation and that TG2 activity was essential for promoting the chemoresistance. These observations clearly conflict with the belief that the physiologic conditions of intracellular environments (low calcium, high GTP, and oxido-reductive state) are not conducive to TG2 in its catalytically active state. Moreover, Yuan et al. [44] recently reported that molecules that are closely related to KCC009 but that lack a TG2 inhibitory effect were able to exert anti-tumor effects similar to those of the parental compound, thus highlighting the need to use caution when interpreting results obtained with TG2 inhibitors.

We directly addressed this problem of TG2 activity by using the catalytically inactive form of TG2 (C277S mutant), and we showed that expression of catalytically inactive TG2 is as effective as is the wild-type TG2 in activating NF-κB activation and downstream events. In stark contrast to previous observations [31], [45], our data demonstrated that irrespective of its catalytic function, TG2-induced activation of NF-κB occurs through its association with the inhibitor IκBα protein. The basal levels of the IκBα transcript in both control cells and TG2-C277S-expressing cells in our study were comparable (data not shown), suggesting that low expression of IκBα protein in TG2 expressing cells is probably related to its destabilization and rapid degradation via a non-proteasomal pathway. We also found the p65/RelA subunit of NF-κB is found associated in complex with TG2, both in the cytosolic and nuclear compartments. Importantly, the TG2/p65 complex could be effectively recruited to a cognate NF-κB binding site in the *HIF-1α* promoter and lead to its transcriptional regulation. At this stage, we do not understand the significance of the TG2 interaction with p65/RelA in regulating the expression of HIF-1α and possibly other NF-κB target genes. It is likely that such interaction may dictate functional regulation of NF-κB in terms of recruiting the co-activators or repressors to the promoter site of target genes. Indeed, multiple post-translational modifications such as phosphorylation, acetylation, or methylation are known to affect this ability of NF-κB [37], [46].

Our observation that TG2-induced activation of NF-κB results in a high basal expression of HIF-1α is significant. Like TG2, HIF-1α expression is considered a negative prognostic factor because of its ability to promote chemoresistance, angiogenesis, invasiveness, metastasis, resistance to cell death, altered metabolism and genomic stability [47]. Initially, HIF-1α was thought to mediate the response only to hypoxia because of its instability under normoxic conditions However, the HIF-1α protein has recently been shown to be upregulated under normoxia in response to certain hormones, growth factors, and cytokines [37], [47]. Our data show for the first time that TG2 can regulate HIF-1α transcription by activating NF-κB. Indeed, inhibition of either TG2 or p65/RelA attenuated HIF-1α expression. A recent study observed a similar effect of TG2 on HIF1α accumulation [48]. In that study, the authors argued that TG2-catalyzed polymerization of the von Hippel-Lindau (VHL) protein resulted in its rapid degradation and depletion [48]. They also suggested that the TG2-induced depletion of VHL stabilized and increased the expression of HIF-1α. In contrast to their methods, we used the catalytically inactive form of TG2 and observed a marked increase in both HIF-1α protein and mRNA expression. It is possible that the transcriptional regulation of HIF-1α by TG2 and NF-κB may act in concert with increased stabilization of the HIF-1α protein. Moreover, we found that TG2-induced NF-κB/HIF-1α were indispensible for induction of the EMT, as suggested by the dependence of Snail, Twist and Zeb1 expression on TG2-induced HIF-1α.

Taken together, our data show for the first time a direct link between TG2, NF-κB, HIF-1α, and EMT. Because these pathways are known to promote the aggressive phenotype in cancer cells, we propose that aberrant expression of TG2 represents an important mechanism in progression of breast cancer. Therefore, in-depth understanding of TG2-regulated pathways may offer promising intervention strategies for preventing the progression of breast cancer to metastatic breast disease.

## Materials and Methods

### Cell lines, Vectors, and Reagents

We grew and maintained immortalized human mammary epithelial (MCF10A), breast cancer MCF7, and drug-resistant MCF7-RT cells as previously described [20], [24], [30]. The full-length TG2 (TG2-WT) and its catalytically inactive mutant (TG2-C277S) form were subcloned into a pCDH lentiviral vector (System Biosciences) from a pcDNA3.1 vector as previously described [24]. The lentiviral particles containing control- or TG2-specific shRNA sequences were purchased from Santa Cruz Biotechnology. We stably transfected the MCF10A cells with a vector alone or a vector containing WT or C277S and R580A mutant forms of TG2 as described previously [24]. Briefly, stable clones were selected by growing transfected cells in a puromycin-containing medium (1 µg/mL). Multiple clones were used to rule out potential clonal effects. All experiments were performed between passage 3 and 10. For transient transfection, SignalSilence p65-specific siRNA, control siRNA and antibody against GAPDH and Phospho-p65 (ser536), were purchased from Cell Signaling Technology. Anti-TG2, anti-Snail, and anti-β-actin antibodies were purchased from Abcam; anti-IκBα antibodies were purchased from Imgenex Corporation; and anti-p65/RelA, anti-ZEB1, and anti-Twist antibodies were purchased from Santa Cruz Biotechnology. Lipofectamine 2000, Oligofectamine, and the Stealth RNAi (negative control) were obtained from Invitrogen (Carlsbad, CA). For TNFα treatment, cells were cultured in medium and treated with 10 ng/mL of recombinant-TNFα.

### Immunoblotting

For Western blots, cells were lysed on ice in NP-40 lysis buffer (50 mM Tris-HCl, 150 mM NaCl, and 0.5% NP-40, pH 7.5). Forty micrograms of total protein from each sample was resolved on a 10–12% SDS-polyacrylamide gel with running buffer and transferred onto nitrocellulose membranes. The membranes were then probed with the appropriate primary and horseradish peroxidase-conjugated secondary antibodies.

### TG2 Activity

The catalytic activity of TG2 (transamidation reaction) was studied in cell lysates by determining the calcium-dependent conjugation of primary amine substrate 5-(Biotinamido) pentylamine (BPA; Pierce Biotechnology) into cellular proteins. After overnight culturing, cells at 80–90% confluence were washed and lysed in Tris-buffered saline (50 mM, pH 7.8), containing 10 mM β-mercaptoethanol and protease inhibitor cocktail (Sigma-Aldrich). Equal amounts of cell proteins (50–80 µg) were transferred into glass tubes in duplicates, and 5 mM calcium chloride or EDTA was added. The reaction was initiated at 37°C by adding 5 mM BPA. After 30 minutes, the reaction was stopped by adding 3× sample buffer. Equal volumes of reaction mixtures were fractionated using SDS-PAGE on 10% gel and electrophoretically transferred onto a nitrocellulose membrane. The membrane was probed with horseradish peroxidase-conjugated streptavidin (Sigma-Aldrich) followed by an electrochemiluminescence reagent (Amersham).

### Nuclear and Cytosolic Fractions

Cytoplasmic fractions were prepared using the NE-PER nuclear and cytoplasmic extraction reagents kit (Pierce Biotechnology). Briefly, cells were harvested and re-suspended in CER 1 buffer (including protease inhibitors), and kept on ice for 10 minutes. Then 5.5% of CERII buffer was added and vortexed vigorously for 10 seconds, followed by centrifugation at 13,000 rpm for 5 minutes. The resulting supernatant was saved as the cytosolic fraction. The insoluble pellet was resuspended in NER buffer and continuously vortexed for 15 seconds every 10 minutes for a total of 40 minutes. After centrifugation at 13,000 rpm for 10 minutes, the resulting supernatant yielded the nuclear extract.

### Immunofluorescence

To evaluate sub-cellular localization of p65/RelA in TG2-transfected cells, we performed immunofluorescence staining of monolayer cell cultures as previously described [20]. Briefly, cells were grown on sterile glass cover slips for 24 hours and treated with TNFα for 30 minutes. Monolayers of cells were then washed with PBS and fixed with 3.7% paraformaldehyde at 37°C for 15 minutes. Fixed cells were permeabilized with 0.1% Triton X-100 for 5 minutes at 4°C, followed by 1 hour incubation at room temperature in blocking solution (5% goat serum in PBS) and then incubation with mouse primary anti-TG2 (clone CUB7401) and rabbit anti-p65 polyclonal antibody (1∶100) overnight at 4°C. After washing, cells were incubated for 1 hour in PBS containing Alexa fluor 610-R phycoerythrin conjugated with goat anti-mouse antibody (1∶200; Molecular Probes, Invitrogen) and Alexa fluor-488 conjugated goat anti-rabbit antibody. Cells were then imaged in the dual-scan mode on a Nikon fluorescence microscope.

### Electrophoretic Mobility Shift Assay

NF-κB activity was determined by electrophoretic mobility shift assay (EMSA) as described previously [24]. In brief, nuclear extracts from 1.5×10^6^ cells were incubated with ^32^P end-labeled NF-κB oligonucleotide probe (5′-**TTG TTA CAA GGG ACT TTC CGC TGG GGA CTT TC**C AGG GAG GCG TGG-3′ [bold-face indicates NF-κB binding sites]) for 30 minutes at 37°C, and the DNA-protein complex formed was separated from the free oligonucleotide on 6.6% native polyacrylamide gels. The dried gels were visualized with a Storm 820 Phosphor imager, and radioactive bands were quantitated using ImageQuant software (GE Healthcare).

### RNA Extraction, RT-PCR, and Quantitative RT-PCR

Total RNA was extracted using a Qiagen mini-RNA isolation kit according to the manufacturer's protocol. For RT-PCR, 2.5 µg total RNA was reverse transcribed to cDNA using an RT2 First Strand kit (SAbiosciences, Qiagen). An equivalent volume (2 µL) of cDNA was used as the template for PCR using gene-specific primers. Quantitative RT-PCR for NF-κB target genes was performed using an RT2 profiler PCR array (SABiosciences) according to the manufacturer's protocol. Relative change was calculated after normalization to GAPDH, β-actin, and 18 s ribosomal RNA.

### Transfection with siRNA/shRNA

Cells (5×10^5^/well) were plated in 6-well plates and allowed to adhere for 24 hours. The next day, the cell monolayers were transfected with 30 nM of p65/RelA-specific or control-siRNAs using oligofectamine (Invitrogen) according to the manufacturer's protocol. Briefly, 2 µL of transfection reagent was added to 100 µL of culture medium, thoroughly mixed, and incubated at room temperature for 15 minutes; 6 µL of siRNA was added to 150 µL of culture medium and then combined with the diluted oligofectamine. The solution was gently mixed, incubated at room temperature for 20 minutes, added to the cell culture, and incubated for the next 48 hours. At the end of the incubation, cell lysates were prepared for further analyses.

For transfection with shRNA, cells (10^4^ cells/well) were plated in 12-well tissue culture plates and allowed to adhere for 24 hours. Monolayers of cells were then transfected with 5 µg/mL polybrene in a culture medium containing 5×10^4^ lentiviral particles with TG2-shRNA or control shRNA sequences, following the manufacturer's protocol. Cells were prepared for further analyses as described above.

### Immunoprecipitation

For immunoprecipitation, cells were washed with ice-cold PBS, collected, and pelleted by centrifugation. Cell pellets were lysed in buffer (50 mM Tris, pH 7.4, 150 mM NaCl, 5 mM EDTA, 0.5% NP-40, protease inhibitor cocktail) and 0.5 mg of proteins was incubated with either anti-p65/RelA or anti-IκBα (2 µg, each) antibody overnight at 4°C. The next day, 30 µL of Protein A/G Plus-Agarose bead suspension was added to 500 µL of reaction buffer and incubated for 1 hour at 4°C. Beads were washed 3 times (1 mL each) with ice-cold lysis buffer and immunoprecipitated proteins were eluted by heating at 95°C for 5 minutes in Laemmli sample buffer [50 mM Tris HCl, pH 6.8, 2% SDS (v/v), 0.001% bromophenol blue, 10% glycerol (v/v), 100 mM dithioerithreitol].

### Chromatin Immunoprecipitation Assay (ChIP)

The ChIP assay was performed using an EZ-Magna ChIP™ kit from Millipore according to the manufacturer's instructions. Briefly, cells were treated with 1% formaldehyde in medium to induce protein crosslinking, lysed in buffer, and sonicated to shear DNA to lengths between 200 and 1000 base pairs. Lysates were centrifuged and supernatants were precleared with Protein A Agarose/Salmon Sperm DNA beads and incubated overnight with either anti-p65 or anti-TG2 antibody (5 µg). Immunoprecipitates were pulled down by Protein Agarose/Salmon Sperm DNA beads and reverse-crosslinked with NaCl. PCR was performed on phenol-chloroform-extracted DNA using primers for the HIF-1α promoter [forward: 5′-cga gga gaa aga gag cag ga-3′; reverse 5′- cgt gct cgt ctg tgt tta gc-3′] flanking the NF-κB binding site (−197/−188 bp) [37]

## Supporting Information

Figure S1 **Supershift EMSA assay for TG2-C277S- induced NF-κB activity.** For details, see the Materials and Methods section.
(TIFF)

Figure S2 **The ability of various TG2 constructs to promote NF-κB activation.** A- NF-κB activity induced in response full-length (TG2-WT), GTP-binding null (TG2-R580A) or catalytically inactive (TG2-S277S) forms of TG2 in MCF10A cells is shown as determined by EMSA. MCF10A-vector cells (Vec), incubated in medium alone or medium containing TNFα (+) served as negative and positive controls, respectively. Drug-sensitive (WT) and -resistant (RT and Dox) MCF-7 sublines, lacking and overexpressing high basal levels of TG2, were also tested for NF-κB activity. B- Western blot analysis for TG2 expression in various cell lines tested for NF-κB activity in A. Membranes were reprobed with anti-β actin antibody to ensure even protein loading in each lane.
(TIFF)

Figure S3 **TG2-induced activation of NF-κB is associated with increased phosphorylation of p65 (Ser 536) subunit of NF-κB.** Immunoblot analysis of phospho-p65 (Ser 536) was performed on cell extracts from MCF10A-Vec, MCF10A TG2-C277S, MCF-7 and MCF-7/RT cells. Membrane was stripped and reprobed with anti-p65 and anti-β-actin antibody to ensure even protein loading in different lanes.
(TIFF)

Figure S4 **Effect of TG2 downregulation on expression of selected NF-κB target genes.** A-Quantitative RT-PCR array showing relative changes in the expression of NF-κB target gene expression in drug-resistant MCF-7 (MCF-7/RT) cells stably transfected with control- or TG2-specific shRNA. Expression of GAPDH, β-actin, and 18S ribosomal RNA was used to normalize variable template loadings. B- Immunoblot analysis was performed to validate the effect of TG2 knockdown on Twist, Snail, and Zeb1 expression in MCF-7/RT cells. Membranes were also probed with anti-TG2 to determine the extent of TG2 downregulation by shRNA.
(TIFF)

Table S1 **TG2-C277S-induced alterations in NF-κB target genes relative to vector-transfected control cells, as determined by real-time PCR array.**
(DOCX)
