# Supplementary figures and images for: Tissue Transglutaminase Constitutively Activates HIF-1α Promoter and Nuclear Factor-κB via a Non-Canonical Pathway
Source: PLoS One. 2012 Nov 19;7(11):e49321. doi: 10.1371/journal.pone.0049321 (PMC3501523; doi:10.1371/journal.pone.0049321)

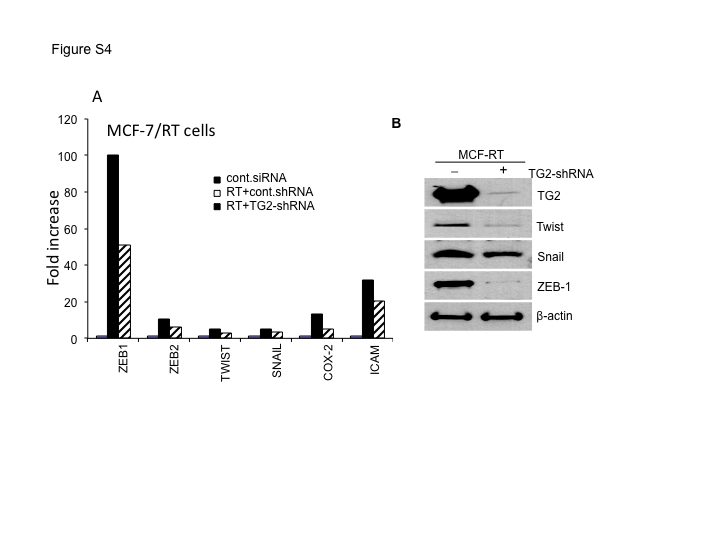

Supplement: Figure S4 — Effect of TG2 downregulation on expression of selected NF-κB target genes. A-Quantitative RT-PCR array showing relative changes in the expression of NF-κB target gene expression in drug-resistant MCF-7 (MCF-7/RT) cells stably transfected with control- or TG2-specific shRNA. Expression of GAPDH, β-actin, and 18S ribosomal RNA was used to normalize variable template loadings. B- Immunoblot analysis was performed to validate the effect of TG2 knockdown on Twist, Snail, and Zeb1 expression in MCF-7/RT cells. Membranes were also probed with anti-TG2 to determine the extent of TG2 downregulation by shRNA. (TIFF) [file pone.0049321.s004.tiff]
