# Supplementary material for: Tissue Transglutaminase Constitutively Activates HIF-1α Promoter and Nuclear Factor-κB via a Non-Canonical Pathway
Source: PLoS One. 2012 Nov 19;7(11):e49321. doi: 10.1371/journal.pone.0049321 (PMC3501523; doi:10.1371/journal.pone.0049321)
Supplement: Table S1 — TG2-C277S-induced alterations in NF-κB target genes relative to vector-transfected control cells, as determined by real-time PCR array. (DOCX) [file pone.0049321.s005.docx]

Table S1

**Target gene Relative fold change**

ZEB1 14.96871966

ZEB2 10.62471062

TWIST1 3.293247078

SNAI1 5.084337819

COX2 4.42633641

IKB 1.538254304

NOS2 0.421957559

MTA1 0.333537032

HOMX1 3.139960519

CDKN1A 0.278391086

CCND1 0.142452574

ICAM1 4.049870974

COPS2 0.670282005
